# Which Future for Neuroscience in Forensic Psychiatry: Theoretical Hurdles and Empirical Chances

**DOI:** 10.3389/fpsyt.2013.00074

**Published:** 2013-07-25

**Authors:** Luca Casartelli, Cristiano Chiamulera

**Affiliations:** ^1^IEB, Geneva University Medical School, University of GenevaGeneva, Switzerland; ^2^Child Psychopathology Unit, Scientific Institute, IRCCS Eugenio MedeaLecco, Italy; ^3^NeuroPsiLab, Department of Public Health and Community Medicine, University of VeronaVerona, Italy

## Neuroscience Data and Forensic Psychiatric Examination: Opportunities, Threats, and Limitations of the Current Debate

Almost all of the world’s legal systems assume that criminal responsibility cannot be established if the defendant was not capable to appreciate the wrongfulness of his/her actions (1). Furthermore, most Western Penal Codes assume that the defendant cannot be convicted if he/she is considered unable to evaluate the circumstances surrounding the anti-juridical fact, and/or if he/she is unable to control his/her urges, impulses, or responses. If a subject lacked these abilities, then courts may use statements such as “not guilty by reason of insanity,” “incapacitated person,” or “(partial) mental insanity” (2). Recent developments of neuroscience in clinical practice have lead to consider the potential role of neuroscience data for assessing criminal responsibility in forensic psychiatric examination (FPE) (3). In the literature, opportunities, threats, and limitations of the introduction of neuroscience in FPE have been widely debated (4, 5). It has been proposed that the use of neuroscience data in FPE may, at least in some circumstances, support the detection of such disabilities. Conversely, it has been also stressed the potential misleading role of neuroscience data in courts.

Traditionally, the debate focused on four major issues:

(a) renowned judgments involving the reference to neuroscience data in FPE (6–8)
(b) the relationship between brain abnormalities and violent behaviors leading to criminal acts:
  * in conduct disorders (e.g., antisocial personality disorder, psychopathy, deficit of self-control) (9–11)
  * following traumatic brain injury (12–14)
  * in sleep/awareness disorders (15–17)
(c) opportunities and limitations of neuroimaging techniques (e.g., Magnetic Resonance Imaging, MRI; functional MRI, fMRI; Positron Emission Tomography, PET) (18, 19), behavioral genetics (20), and lie-detection techniques (21)
(d) Heterogeneity in admissibility criteria of neuroscience data through different legal systems (22, 23). Let’s consider US legal system: the rules governing expert testimony usually refer to “Frye” or “Daubert” approach (24). Furthermore, the crucial but controversial role of Federal Rules of Evidence (notably FRE 401, FRE 403, and FRE 702) in US legal system is a vexing issue in the literature [for a detailed description, (18)]. In many legal system, e.g., in Italy, there is not something like Federal Rules of Evidence and the judge has to evaluate – case by case – evidences presented by experts (e.g., psychiatrist, psychologist, or neurologist). If the interpretation of FRE may sometimes be hard and misleading, no specific criteria for admissibility (except for judge’s decision) may be also extremely dangerous.

Our aim is to highlight the critical issues about the conditions that support – in specific circumstances – the introduction of neuroscience data in FPE. In other words, before advocating or refusing the use of neuroscience data in FPE, we have to clarify the role that these data can have in FPE (i.e., a probative value for forensic assessment).

Firstly, we shortly hint at classical cognitive paradigms and their limitations. Secondly, we show that most of the Western Penal Codes are shaped assuming a sort of dualist model of human cognition. Finally, we describe potential misleading implications of neuroscience data endorsed in a dualist legal framework. Even if no neuroscience data can definitively discriminate neither between crime nor a mental defect, and neither between culpability nor innocence, we suggest that the introduction of neuroscience in FPE may be – in specific circumstances – useful and compelling.

## Theoretical Hurdles and Empirical Chances: Which Implications for Forensic Settings?

Classically, a dualist model of human cognition assumes a clear distinction between two different substances: the mind and the body (25, 26). In such model, the mind has a direct control on bodily parts. In other words, the mind (roughly speaking, “mental states”) would control and manage the body (roughly speaking, “bodily acts”) through a sort of direct causal relationship. Recent advances in brain researches have arisen new theoretical dilemma. There is a widespread agreement, both in philosophy (27, 28) and cognitive neuroscience (29, 30), that underlines the incompatibility between neurobiological data and dualist conceptual framework. It is assumed that if neuroscience data are introduced in a dualist model, then misleading consequences could be taken for granted. Recent studies seem demand a break with traditional approaches to cognition.

Classical cognitivism and many paradigms in philosophy of mind consider mental operations clearly detached from the body, supporting the analogy between higher cognitive functions and specific computations. Following such perspectives, the content of mental representations should be explained in terms of pure mental states (i.e., intentions, beliefs, and desires). From a different point of view, an embodied perspective claims that many features of cognition deal with (causally or even constitutively) physical body (31, 32). It is still controversial the role of mental representation in embodied approaches. Some scholars have proposed the concept of “body representation” (such as a distinctive class of mental representation), claiming that they are the most promising notion to promote an embodied approach to social cognition (33). Others have proposed the notion of “embodied simulation,” arguing that one can understand others by reusing one’s own mental states or processes such as representations formatted in terms of bodily format (34). Both topics are hotly debated in the literature (35, 36).

Experimental studies seem support the idea that a new theoretical framework of human cognition has to be drawn. Let’s consider an interesting case. Mirror neurons are a distinct class of visuomotor neurons discovered in the ventral premotor cortex of macaque monkeys (37). More recently, evidences of the existence of a mirror neurons system in humans come from non-invasive neurophysiological techniques and neuroimaging studies (38, 39). Anatomically, two main cortical networks with mirror properties have been described in humans: the first one is the parieto-frontal mirror system (formed by the parietal lobe and the premotor cortex plus the caudal part of the inferior frontal gyrus); the second one is the limbic mirror system (formed by the insula and anterior mesial frontal cortex) that provides a direct understanding of emotions of others without higher order cognitive mediation (40). The role of parieto-frontal mirror circuit is hotly debated in the literature. The major interest concerns its functional role in action/intention understanding, in imitation, and more generally in social cognition. Even if many mechanisms may mediate the understanding of other’s behavior, a compelling view supports the hypothesis that parieto-frontal mirror circuit is the only mechanism that allows to understand the action of others “from the inside” (41). The case of mirror mechanism is emblematic; it seems support the hypothesis that recent advances in neuroscience demand a new theory of action execution, of action perception, and more generally a new model for human cognition. Such theoretical considerations are not without practical consequences: the value of neuroscience data can be misunderstood if we endorse them into a misleading perspective. In the next part, we show how and why these considerations may concern also FPE.

## Dualist Model in the Forensic Field: Which Problems, if Any, with Neuroscience Data

To assess criminal responsibility, Western Penal Codes usually consider two specific elements. The individual’s body that has materially performed a crime (res extensa, latin for matter of facts) and individual’s mind has taken the decision to commit the crime (res cogitans, latin for mental capabilities). As above reported, it is also scheduled the possibility that a “mental defect” assessed in FPE restrains the possibility to attribute criminal responsibility. This model not only seems remind the dualist framework but it also reflects the way in which forensic psychiatrists usually are called to make assessments about mental capability. Let’s consider the Italian case. Italian Penal Code assumes a clear distinction between mind and body, internal mental states, and external bodily acts. At trial, an Italian judge asked a psychiatrist or psychologist an assessment about “mental capability” of the charged (Codice Penale, art. 88–89) (42), that is to distinguish between crime (i.e., naively “badness”) and mental defects (i.e., naively “madness”). However, there are some problems if we want to improve the assessment in FPE introducing also neuroscience data. “Mental defect” is an extremely ambiguous definition: does “mental defect” include also neurobiological data or, more classically, does it exclusively refer to a behavioral model? Moreover, the Italian judge asked the expert to clarify the “influence” (i.e., they intend causal link or deterministic relationship) between such as “mental defect” and mental abilities during the criminal act. In a dualist perspective, where mind (i.e., “mental states”) directly control the body (i.e., “bodily acts”), it is possible to suppose a causal link between “mental defect” and specific behavior (e.g., aggression). From the perspective of clinical neuroscience, this is a misleading supposition. In fact, experimental data cannot retrospectively assess such as deterministic relationship, given that it can indicates just a statistic-laden correlation. Neuroscience cannot definitively discriminate neither between crime and a mental defect, neither between culpability and innocence.

## Conclusive Considerations

If it is widely assumed that neuroscience data cannot be profitably endorsed in a dualistic perspective, and if it is possible to show that the most of Western Penal Codes are shaped on a dualistic model, then there are compelling reasons to encourage a new perspective also in the forensic field. As above reported, it is not a theoretical proposition without practical consequences. We have stressed the idea that empirical data cannot directly assess criminal responsibility. Neuroscience data may be useful – in specific and limited circumstances – to give aid to traditional forensic assessment for mental capabilities. Clearly, not all neuroscience data may assume the same explanatory value, and not all data may be useful in FPE. The renowned debate on fMRI data is illustrative. On one side, researches both in biological psychiatry and neuropsychology have been considerably improved by fMRI studies (43, 44); on the other side, the effective implications of such studies are still controversial in clinical practice (45) and also in different research fields (e.g., economic behaviors, forensic settings, moral development, etc.) (18, 46, 47). Nevertheless, the debate on the probative value of specific neuroscientific techniques overcomes the aim of this work.

In conclusion, we suggest that the preliminary condition to introduce neuroscience data in FPE is the assumption of a new perspective overcoming classical dualist models. Such new perspective permits to rule out misleading assumptions (i.e., the deterministic link between “mental defect” and specific behavior). Noteworthy, it is a necessary but not sufficient condition to introduce neuroscience data in FPE, given that such data has to be evaluated case by case.
